# The case for conducting first-in-human (phase 0 and phase 1) clinical trials in low and middle income countries

**DOI:** 10.1186/1471-2458-11-811

**Published:** 2011-10-18

**Authors:** Lydia Kapiriri, James V Lavery, Peter A Singer, Hassan Mshinda, Lorne Babiuk, Abdallah S Daar

**Affiliations:** 1 Department of Health Aging and Society, McMaster University, 1280 Main St. W, Hamilton, Ontario, L8S 4M4, Canada; 2 Centre for Research on Inner City Health and Centre for Global Health Research, Keenan Research Centre in the Li Ka Shing Knowledge Institute of St. Michael's Hospital & University of Toronto 209 Victoria St, 3rd floor, Toronto, Ontario, M5B 1C6, Canada; 3 Centre for Global Health MaRS Centre, South Tower, 101 College Street, Suite 406, Toronto, Ontario, M5G 1L7, Canada; 4 Tanzania Commission for Science and Technology, P.O. BOX 4302, Dar es salaam, Tanzania; 5 University of Alberta, University of Alberta, 1-20 University Hall, Edmonton, Alberta, T6G 2J9, Canada

## Abstract

**Background:**

Despite the increase in the number of clinical trials in low and middle income countries (LMICs), there has been little serious discussion of whether First in Human (FIH; phase 0 and phase 1) clinical trials should be conducted in LMICs, and if so, under what conditions. Based on our own experience, studies and consultations, this paper aims to stimulate debate on our contention that for products meant primarily for conditions most prevalent in LMICs, FIH trials should preferably be done first in those countries.

**Discussion:**

There are scientific and pragmatic arguments that support conducting FIH trials in LMIC. Furthermore, the changing product-development and regulatory landscape, and the likelihood of secondary benefits such as capacity building for innovation and for research ethics support our argument. These arguments take into account the critical importance of protecting human subjects of research while developing capacity to undertake FIH trials.

**Summary:**

While FIH trials have historically not been conducted in LMICs, the situation in some of these countries has changed. Hence, we have argued that FIH should be conducted in LMICs for products meant primarily for conditions that are most prevalent in those contexts; provided the necessary protections for human subjects are sufficient.

## Background

Despite the sustained proliferation of clinical trials in low and middle income countries (LMICs), [1] there has been little examination of whether First in Human (FIH; phase 0 and phase 1) clinical trials should be conducted in LMICs, and if so, under what conditions. Most of the discussion has focused on later phases of clinical trials. The purpose of this paper is to stimulate debate on the merits of FIH trials in LMICs: the default option should be to do the trials where the relevant health conditions present the greatest public health challenge, if, and only if, the necessary capacities exist for participant safety and scientific rigor. We argue, further, that the default presumptions against performing FIH trials in LMICs may be quietly impeding progress in the evolution of the very capacities that these trials require.

Interest in FIH clinical trials has grown recently, in part because of the disastrous experience of the phase I clinical trial of the superagonistic anti-CD28 antibody TGN1412 in the United Kingdom. The drug was developed with the intention to stimulate a specific kind of T-cell while at the same time controlling the production of other T-cells in order to suppress the immune system. In this trial, six healthy volunteers developed a cytokine release syndrome with multi-organ failure and required intensive care [2,3]. There were mistakes made in that case, and some of the key lessons have been translated into more stringent rules and new guidelines [3-5].

FIH trials are studies where an investigational medical product (drug, vaccine, medical device, etc), previously developed and assessed through *in vitro *or animal testing, or through mathematical modeling, is tested on human subjects for the first time [4,5]. In drug development, such trials involve administering single low, sub-therapeutic doses to a small number of healthy volunteers (10 to 15) to gather preliminary data on the product's pharmacokinetics and pharmacodynamics. These trials help researchers identify the drug candidate with the best pharmacological parameters to take forward for further development and which one to leave out. Traditionally, these early phase trials have been conducted in high income countries (HICs). Until recently they were very rarely, if ever, performed in LMICs even for conditions that are most prevalent in those countries. Why haven't FIH trials been more common in LMICs?

The main historical reasons have been the lack of research and clinical infrastructure, inadequate institutional capacity, and weak regulatory agencies in many LMICs. This has made it difficult for product developers, usually large multinational pharmaceutical companies, and regulatory agencies in developed countries to achieve the necessary standards of safety for trial participants, and clinical and scientific rigor in LMICs [6]. LMICs themselves have often relied on product registration processes by regulatory authorities in developed countries [6]. At the same time, regulatory authorities in developed countries often lack knowledge of the diseases, including many of the so-called Neglected Tropical Diseases (NTDs), and the local conditions most prevalent in LMICs, making it possible that they will make judgments based on inappropriate risk-benefit assessments [7], potentially to the detriment of populations in LMICs. Another reason is that Western companies, which currently develop most new and innovative products, have considered it risky to conduct FIH trials in LMICs for various reasons, including the inability to discriminate adverse events (AEs) caused by the investigational product from the generally much higher frequency of all types of undiagnosed symptoms and untreated morbidities, the cultural obstacles to undertaking autopsies and the fear that they may be perceived to be exploiting vulnerable persons in these countries [8]. The fact that so few companies do FIH trials in low income countries leads to a situation where others are reluctant to start, and this makes more trial sponsors also reluctant to start. The net result is a general presumption against FIH trials in LMICs.

This situation is beginning to change. This shift presents an opportunity to engage in a serious debate about the basic logic of site selection for FIH trials. Here, we are focused on FIH trials for diseases that present important public health challenges for LMICs, including the rapid emergence of a wide range of chronic diseases [9]. In particular, we agree with the ethical argument that trials should be responsive to the health needs of host communities in the sense argued by London and Kimmelman, that is, if they are "part of a program of inquiry that will expand the capacity of health-related social structures in the host community to meet urgent health needs."[10]. For example, it makes little sense to perform FIH trials of malaria vaccines in the United States today, as has happened recently with the live attenuated sporozoite vaccine developed by Sanaria [11], when malaria transmission stopped in the US after World War II [12], but it continues to kill about 1 million children a year in sub-Saharan Africa [13].

There are also strong scientific and pragmatic arguments. The diseases in question are most prevalent in LMICs, where the epidemiology, health services, social determinants of health, compliance patterns, co-morbidities and the genetic make-up of the population also have a bearing on the way in which the health products will ultimately be used and hopefully achieve the desired outcomes [14]. There is evidence that, mutations in genes coding for drug-metabolizing enzymes may result in drugs being metabolized differently in different populations [15], affecting the response and AEs to drugs and vaccines [16]. The gastrointestinal microbiome may also make a difference. Individuals with gastrointestinal infections have been found to respond differently to medical products, for example the live polio vaccine [17]. Thus, testing drugs and vaccines in developed countries, (whether in early or late phase trials), may give misleading results.

An important consideration also relates to the need to conduct both early and late phase clinical trials among subjects who have the target disease. The ethical issues here may need further reflection, but scientifically, there are circumstances and arguments where it would be beneficial to conduct such studies among the patient population. For example, if there's need to evaluate a new product that cannot be assessed in healthy individuals for some reason, including ethical considerations, then it may be necessary to recruit patients with the target diseases. In other cases, it may be difficult to extrapolate the results from healthy individuals to a patient population. In such instances, the results pertaining to a new product maybe either over- or under-estimated [18,19]. In these examples, it can be extrapolated to product development for conditions that are most prevalent in low income countries, and argued that it would be reasonable for early stage trials to be conducted in these countries where the relevant diseases are most prevalent and it is possible to recruit patients.

The need to reconsider where FIH trials are done first is becoming even more salient as LMICs push to expand their involvement in all phases of drug development, primarily as a source of revenue. More important, though, is the fact that the pipeline of products for conditions that occur predominantly in LMICs is improving significantly [20]. Major philanthropies such as the Bill & Melinda Gates Foundation fund discovery research directly through programs such as the Grand Challenges in Global Health initiative [21], or indirectly through many product-developing Public-Private Partnerships (PPPs) [22], Similarly, large multinational pharmaceutical companies have begun, on their own or through PPPs, to develop more health products for NTDs and for diseases most prevalent in LMICs. Countries such as India [23], China [24,25], and Brazil [26], now have their own strong and growing pharmaceutical and vaccine manufacturing industries- indeed half of childhood vaccines administered throughout the world by UNICEF are made by one company in India [23].

An important underlying concern in this discussion is, of course, the safety of human research volunteers and the potential for exploitation of vulnerable populations in LMICs (or indeed anywhere else in the world). By definition, the risks in FIH trials, particularly for phase 0, are not known for sure: they could be non-existent, low or, as in the TGN1412 case, high [2,3,22,27,28]. In addition to posing risks to the participants, FIH trials that do not completely take into account the design of, and data from, preclinical trials may not provide the necessary information to adequately evaluate the results of FIH trials [5]. TGN1412 and other FIH trials in HIC have shown us clearly that risks are inherent in these trials and not unique to LMICs, and so we must be careful not to impose more stringent standards on LMICs (especially those which meet the necessary GCP conditions) than we do on HIC.

## Discussion

### The necessary conditions exist

The United States Department of Health and Human Services Office of the Inspector General (OIG) report of June 2010 documents the increasing number of clinical trials being conducted in LMIC [29,30], including a few FIH trials [10,31]. UNAIDS also reports an increase in various FIH HIV vaccine and microbicides trials in Kenya, South Africa, Thailand and Uganda [32]. Furthermore, the U.S. National Institutes of Health (NIH) clinical trials registry also reflects an increasing number of phase I trials in LMICs most of the increase having been registered in the most recent past 5-10 years [33] (Table 1).

**Table 1:** Number of Phase 1 trials in low income countries as registered by the NIH (2010)

Region	Number of Phase I trials registered
Africa	103 (0 registered between 1995/2000)
Central America	84
East Asia (including Japan)	659
Middle East	267
South America	128 (0 registered between 1995/2000)
South Asia	94 (81 registered in India)
South East Asia	120

Some commentators argue that that it is too risky to conduct FIH trials at all in LMICs, since the necessary conditions do not exist to ensure high technical standards and the safety of research participants [34,35]. We disagree with this assessment and believe this presumption unfairly treats all LMICs alike, even in the face of growing evidence of rapid capacity development in many LMICs. A number of initiatives to support clinical trials, including FIH trials, in some LMICs, have had an impact. In India, China, and Brazil, for example, Good Manufacturing Practice (GMP) and Good Clinical Practice (GCP) standards have improved dramatically [6]. These standards often bear a close relationship to the quality of healthcare services more generally. There are new hospitals/institutions, some of them in the private sector, that are widely understood by clinical trial specialists in the West to have the same capacity as clinical trial centers in the U.S to conduct FIH trials safely and to high scientific standards [31]. How did these improvements come about?

Having accepted that standards must be the same for all countries, LMICs sought assistance in infrastructure development and training that would facilitate implementation of a single set of internationally-harmonized GCP standards- a goal that led the World Health Organization (WHO) to develop its 2002 GCP Handbook and to embark on a series of educational/training programs in GCP in LMICs [36]. The United States Food and Drug Administration (FDA) regulations for the acceptance of data from non-U.S. studies are in fact linked to these internationally harmonized GCP standards. Indeed, FDA now assists LMICs to build capacity to review and inspect clinical research within their own legal jurisdictions, as a way to improve the quality of clinical trial data that might ultimately be submitted to FDA [37]. Consequently, regulatory capacity is slowly being enhanced not only in the emerging economies of India and China, where FDA itself has set up offices [38], but also in countries in sub-Saharan Africa [5,20].

Low and middle income countries cannot depend on others for oversight of their clinical trials. The FDA, for example, despite its good intentions, has been found to lack the capacity "to effectively oversee clinical trials conducted in LMICs" [1,6]. Particularly for the early phase trials, the FDA has been reported to be unaware that the trials are even going on [6], making it impossible for them to provide the necessary oversight. FDA's contribution should be in helping to build local capacity for oversight or emulating the European Medical Agency (EMEA), which has partnered with the WHO to develop detailed guidelines intended to provide a mechanism for licensing products of major public health interest for LMICs which are not expected to be licensed in the EU. Under this partnership, EMEA evaluates data on the quality, safety and efficacy of the product contained in the application in collaboration with the WHO, before issuing a scientific opinion regarding the benefit-risk ratio of the product [39]. These initiatives have succeeded in ensuring that some institutions in LMICs now have the capacity to conduct FIH trials and to provide leadership and experience in the development of rigorous and responsive home-grown clinical trials programs.

### Secondary benefits

Conducting FIH trials in low income countries in accordance with international regulatory standards should also drive capacity building for local ethical review, facilitate health care infrastructure development, increase economic activity by encouraging research into more innovative products, and reduce the culture of dependency on developed countries [6,40,41]. As noted, there are many late-phase clinical trials taking place in LMICs, helped by initiatives to strengthen clinical trial sites [42]. These include initiatives associated with the European and Developing Country Clinical Trial Partnership (EDCTP) [43], the Malaria Clinical Trials Alliance, the African Malaria Intervention Network (AMANET] [44], the African Vaccine Regulatory Forum (AVAREF), as well as initiatives to strengthen local training institutions such as Uganda's Mulago Hospital HIV research centre of excellence, which helps provide research training for the Sub-Saharan Africa region [45]. Many of these initiatives developed in response to the proliferation of later phase clinical trials in LMICs, and many researchers from LMICs believe the only way their countries will improve their capacity to conduct and regulate FIH trials is by doing so within their home contexts [41].

We recognize the challenges of regulatory and other deficiencies in those countries that have not already caught up. We neither wish to trivialize the importance of protecting human subjects, nor to argue that all countries are now ready to do FIH trials. But we believe that many countries now have the necessary capacity, or could acquire it quickly, and that improving the necessary conditions everywhere is good for research and good for health. We acknowledge that clinical research in low income countries has a mixed history and in many instances has not enhanced equity. But this is true as well in some high income countries. Hence, we believe that the transition to conducting more FIH trials in low income countries, while also calling attention to deficiencies health systems and regulatory capacity, could help to improve standards, particularly if taking more ownership of trials and product development--as is already occurring throughout the developing world [46,47]--would allow for less reliance on developed countries.

### The way forward: a role for both the developed countries and LMICs

We believe that trial sponsors, ethics review committees, regulatory agencies and other stakeholders should work together to develop explicit policies and programs that would help advance LMICs' ability to conduct high quality clinical trials, including specific guidance and capacity for FIH trials. Hence, in the absence of well developed regulatory and science funding policies and legal frameworks and clinical capacity, all these players will have to get on the same page to develop a conducive environment, emphasizing safety, clinical excellence and scientific excellence.

There will increasingly be products meant specifically for the developing world, manufactured in the developing world, that are of immense value locally, and that will unlikely ever to be used in the developed world. A change in mind-set is needed. LMIC regulatory agencies must prioritize improvements of their own regulatory regimes for these circumstances and begin to reduce their reliance on the FDA or EMEA for prior approval of products before they have the courage to approve them locally. In order to benefit from local experience and be able to benefit more from capacity-building assistance from donor communities, and to negotiate more effectively, it would be wise to coordinate and harmonize local regulatory approaches at a regional level. In East Africa, for example, this could be through the East African Community that links Tanzania, Kenya, Uganda, Rwanda and Burundi. Indeed, there is already a move to establish an East African Medicines and Food Safety Commission [48].

Current thinking about research ethics review and oversight in LMICs is shifting from an emphasis on training and ethical principles to a deeper account of the systems requirements to sustain effective review and oversight [49]. This thinking might offer some direction about what types of capacity-building will be required to enhance FIH trials in LMICs [50]. The capacity for training ethicists and establishing ethics review boards, which have increased significantly in the past decade, could be strengthened even further through creative programs such as the one developed by the NIH Fogarty International Centre [50], which has spawned dozens of local training programs around the world such as those in India [51], Pakistan [52] and South Africa [53]. Such initiatives could add FIH trial oversight to their curricula. We expect that some of the most advanced emerging economies will continue to lead, but the key will be to harness the lessons from these countries in terms of what capacity is needed in science, policy and regulation, etc., so that other less well developed countries will have a path to follow.

### Summary

We have argued that while FIH studies have historically not been done in LMICs, the situation in these countries has changed, and some of the reasons for not conducting FIH in these countries, no longer hold true. Hence, for products meant primarily for conditions that are most prevalent in LMICs, more FIH trials should be performed in LMICs, provided the protections are at least equivalent to those provided in similar trials in developing country clinical settings. While some countries in the developing world already have the capacity to conduct FIH trials, to make this happen on a larger scale will require enhanced capacity in regulatory oversight, health care infrastructure, clinical research infrastructure, and ethics review in those countries where these have not yet reached the necessary standards. Since the advantages extend to both the developing and developed worlds, we argue that all should work cooperatively to address those impediments that currently discourage FIH trials from being done in the developing world.

## Competing interests

The authors declare that they have no competing interests. The funders had no role in study design, data collection and analysis, decision to publish, or preparation of the manuscript

## Authors' contributions

All authors participated in the development of the manuscript. LK, ASD, JL, PS contributed to the development of the main ideas in the paper. HM & LB contributed to further development and refinement of the ideas in the paper. All authors contributed to finalizing of the submitted manuscript.

## Pre-publication history

The pre-publication history for this paper can be accessed here:

http://www.biomedcentral.com/1471-2458/11/811/prepub
